# The Effect of Primary Cancer Cell Culture Models on the Results of Drug Chemosensitivity Assays: The Application of Perfusion Microbioreactor System as Cell Culture Vessel

**DOI:** 10.1155/2015/470283

**Published:** 2015-01-14

**Authors:** Chia-Hsun Hsieh, Yi-Dao Chen, Shiang-Fu Huang, Hung-Ming Wang, Min-Hsien Wu

**Affiliations:** ^1^Division of Hematology-Oncology, Department of Internal Medicine, Chang Gung Memorial Hospital at Linkou, Taoyuan 333, Taiwan; ^2^Department of Chemical and Materials Engineering, Chang Gung University, Taoyuan 333, Taiwan; ^3^Graduate Institute of Biochemical and Biomedical Engineering, Chang Gung University, Taoyuan 333, Taiwan; ^4^Department of Otolaryngology-Head and Neck Surgery, Chang Gung Memorial Hospital, Chang Gung University, Taoyuan 333, Taiwan; ^5^Division of Hematology-Oncology, Department of Internal Medicine, Chang Gung Memorial Hospital, Chang Gung University, Taoyuan 333, Taiwan

## Abstract

To precisely and faithfully perform cell-based drug chemosensitivity assays, a well-defined and biologically relevant culture condition is required. For the former, a perfusion microbioreactor system capable of providing a stable culture condition was adopted. For the latter, however, little is known about the impact of culture models on the physiology and chemosensitivity assay results of primary oral cavity cancer cells. To address the issues, experiments were performed. Results showed that minor environmental pH change could significantly affect the metabolic activity of cells, demonstrating the importance of stable culture condition for such assays. Moreover, the culture models could also significantly influence the metabolic activity and proliferation of cells. Furthermore, the choice of culture models might lead to different outcomes of chemosensitivity assays. Compared with the similar test based on tumor-level assays, the spheroid model could overestimate the drug resistance of cells to cisplatin, whereas the 2D and 3D culture models might overestimate the chemosensitivity of cells to such anticancer drug. In this study, the 3D culture models with same cell density as that in tumor samples showed comparable chemosensitivity assay results as the tumor-level assays. Overall, this study has provided some fundamental information for establishing a precise and faithful drug chemosensitivity assay.

## 1. Introduction

Chemotherapy is a kind of cancer treatments in which chemical substances are utilized to kill cancer cells in human body. Currently, the decision of a chemotherapy regimen is still based on the empirical information from clinical trials in patients which ignores biological individuality of tumor [1]. In fact, the therapeutic effects of anticancer drugs to cancer cells exhibit high degree of variation [2] because individual patient's tumor is genotypically and phenotypically different [3]. For a more personalized chemotherapy, therefore, an* in vitro* chemosensitivity assays is required to evaluate which anticancer drugs the patient's cancer cells will respond to. This can assist doctors to tailor a chemotherapy regimen for individual patients.* In vitro* anticancer drug chemosensitivity assays mainly involve the basic procedures including (1) isolation of cancer cells from a tumor sample, (2) incubation of cancer cells with anticancer drugs, (3) evaluation of cancer cell viability, and (4) interpretation of the results [1].

For most cell-based assays (e.g., drug chemosensitivity assays), static cell culture models [4, 5], where the culture medium is virtually supplied in a manual and batch-wise manner, were commonly adopted. Nevertheless, this could lead to a fluctuating culture condition [6] that could in turn hamper the precise quantification of the link between the drug conditions tested and cancer cells' response. Moreover, most of the conventional cell culture models are relatively large in scale, which could therefore require larger number of cells for a cell-based assay. In drug chemosensitivity assays, however, the clinical tumor samples harvested and thus the cancer cells isolated are normally limited. Therefore, the isolated primary cancer cells generally need to be expended in number for the subsequent cell-based assays. Nevertheless, the expansion process of cell number (e.g., cell proliferation on a 2D surface) could possibly alter the cellular physiology [7] and in turn might affect the faithfulness of the following chemosensitivity assays. In addition, the cell culture conditions in a relatively large cell culture scale might not be regarded as homogenous mainly due to the chemical gradient phenomenon existing in the cell culture system. Such poorly defined culture conditions could restrict the precise quantification of the link between cellular responses and anticancer drug conditions. To tackle the above technical issues, more recently, perfusion-based microscale bioreactor systems were actively proposed for various cell-based assays [6, 8–10] by which a stable and well-defined culture condition can be achieved due to the continuous medium perfusion format and miniaturized cell culture scale [6, 8].

For the most drug chemosensitivity assays [11–13], moreover, two-dimensional (2D) monolayer cell cultures are commonly used, where the cancer cells attach, spread, and grow on a surface. Such a cell culture model has been widely adopted in life science-related research for more than a hundred years. This is primarily because of its simplicity in terms of the cell culture preparation and the subsequent microscopic observation of cell culture. Nevertheless, 2D culture conditions might not well simulate the* in vivo* microenvironments surrounding biological cells since cells inhabit environments with very 3D features [14]. It has been recognized that cancer cells in a 2D culture environment differ physiologically from those in a 3D environment [15]. In addition to the conventional 2D cell culture model, spheroid culture models, in which cells self-aggregate to form sphere-like 3D cell clusters, are regarded as excellent models for tumor tissues [16]. Due to their 3D nature, they are believed to provide a more biologically relevant microenvironment than 2D monolayer cultures [17]. Spheroid culture models are thus widely utilized in various cancer cell researches [18, 19].

As aforementioned, cells inhabit environments with very specific 3D features in animal tissues. In their native 3D environment, mammalian cells are subject to not only various biological cues such as soluble signaling molecules, but also cell-to-cell interactions and mechanics and dynamics of the surrounding extracellular matrix (ECM) [20]. All these biological signals may determine the fate of cells to undergo proliferation, differentiation, or apoptosis. Borrowing from the concept of tissue engineering, 3-dimensional (3D) culture models, where the biological cells are encapsulated in a 3D polymeric scaffold, are generally believed to provide a better approximation of the* in vivo* conditions than 2D culture models. Therefore, they could provide a more biologically relevant and thus physiologically meaningful culture condition for cell-based assays [21, 22]. Thus far, various 3D cell culture models have been proposed for cancer-related researches [23, 24].

In order to faithfully and precisely investigate the cancer cells' response to anticancer drugs, a stable, well-defined, and biologically relevant cellular microenvironment is needed. In this study, a perfusion-based microscale cell culture system capable of providing a stable culture condition [6] was used for the cell-based chemosensitivity assays. Before the application of drug chemosensitivity assays for guiding future chemotherapy plans, however, there are some fundamental biological issues needed to be addressed. These include what is the result difference of the chemosensitivity assays based on the above-mentioned cell culture models (i.e., conventional 2D, spheroid, and 3D culture models) and which results are closer to the chemosensitivity assay results based on tumor tissue-level assays, an assay model which is more representative of the* in vivo* condition than the cell-based assay counterpart. To more realistically answer the above questions, primary oral cavity cancer cells were used for the assays in this study compared with the cell line models in the previous study [9, 15]. Results revealed that even minor environmental pH change could significantly influence the metabolic activity of the cultured primary cancer cells, demonstrating the importance of stable culture condition for a precise cell-based assay. Moreover, the choice of cell culture formats (e.g., 2D, 3D, or spheroid culture models) might play an important role in the physiology (e.g., metabolic activity or cell proliferation) of the cultured cells. Also the use of different cell culture models could lead to different results of drug chemosensitivity assays. Compared with the tumor tissue-level chemosensitivity assays, moreover, the 3D culture models with same cell density as that in tumor tissue samples showed comparable chemosensitivity assay results as the tumor tissue-level assays. As a whole, this study has provided some fundamental information regarding the influence of cell culture methods on the results of* in vitro* cell-based assays. All these pieces of information are of great importance for establishing a precise and faithful drug chemosensitivity assay.

## 2. Material and Methods

### 2.1. Fabrication and Experimental Setup of Perfusion Microbioreactor System

**Figure 1:**
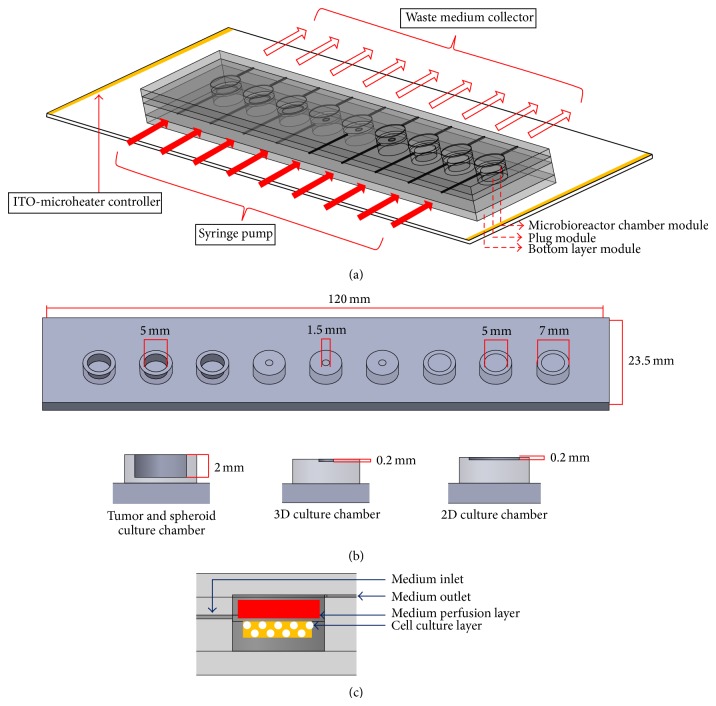
(a) The experimental setup of perfusion microbioreactor system, (b) the schematic illustration of bottom layer module (upper illustration: the topside view, lower illustration: the cross-section view), and (c) the cross-section view of microbioreactor.

In this study, the perfusion microbioreactor system proposed in our previous study [6] was utilized to carry out primary cancer cell-based chemosensitivity assays. Briefly, the perfusion microbioreactor system consists of a microbioreactor chamber module, a plug module, and a bottom layer module (Figure 1(a)). The microbioreactor chamber module is composed of 9 cylindrical microbioreactor chambers with each microbioreactor having the same format as the well of a standard 96-well microplate (D: 7 mm; H: 7 mm). The plug module (Figure 1(a)) contains 9 columns, which are able to plug up the 9 microbioreactor chambers accordingly to form multiple closed systems for perfusion cell culture. Similar to the plug module, the bottom layer module (Figure 1(b)) contains 9 columns to plug up the 9 microbioreactor chambers at the bottom side accordingly. In this work, each column on the bottom layer module not only functions as a “stopper” to seal each cylindrical microbioreactor chamber in each column (Figure 1(b)) but also acts as the compartment to accommodate the cancer cells in 3 different formats for 2D monolayer, 3D, and spheroid cell culture. In addition, such compartment was also used for tumor tissue-based chemosensitivity assay.  Figure 1(c) demonstrates the cross-section view of each microbioreactor. In this study, all the modules in the microbioreactor system were constructed simply by casting of polydimethylsiloxane (PDMS) polymer (Sylgard 184, Dow Corning, USA) on a polymethylmethacrylate (PMMA) mold fabricated using micromachining technique as described previously [6]. For perfusion cell culture purpose, each microbioreactor was perfused with its own separate medium supply through silicon tubing driven by a multichannel syringe pump (KDS 220, KD Scientific Ltd., USA). In this work, the PDMS walls of the microbioreactors were punched using a needle to simply create holes for tubing insertion. In addition, the perfusion microbioreactor system was placed on the surface of a transparent indium tin oxide- (ITO-) based microheater chip to provide a stable thermal condition of 37 ± 0.2°C for cell culture [6, 25]. The entire experimental setup was illustrated in Figure 1(a).

### 2.2. The Isolation of Primary Oral Cavity Cancer Cells

**Figure 2:**
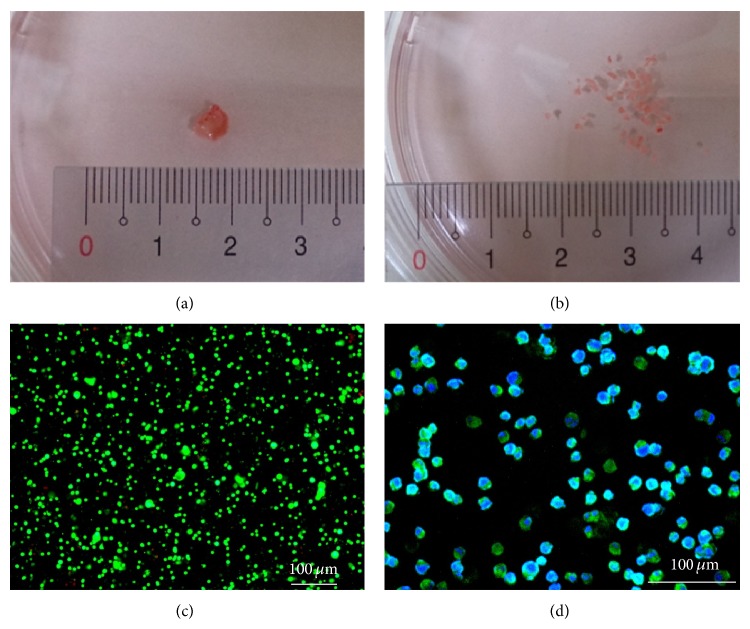
(a) Tumor tissue from an oral cavity cancer patient, (b) diced tumor tissue particles, (c) fluorescence microscopic observation of the isolated primary oral cavity cancer cells (green and red dots represent live and dead cells resp.), and (d) immunofluorescent microscopic images of the isolated primary oral cavity cancer cells (Hoechst dye positive (nucleated cells): the blue dots; EGFR dye positive (cancer cells): the green dots).

The study was approved by the Institutional Review Board of the Chang Gung Memorial Hospital and the informed consent was obtained from all patients (Approval ID: 102-3943B). The clinical tumor tissues were resected from the oral cavity cancer patients in a local medical center. The samples were harvested within 2-3 hrs of surgery. The tumor tissue samples obtained (Figure 2(a)) were first rinsed using phosphate buffered saline (PBS; Invitrogen, Taiwan) and then diced to tiny cubic particles (approximate size:* L*: 0.7 mm;* V*: 0.343 mm^3^) (Figure 2(b)) using a surgical scalpel blade. All process was carried out aseptically. The prepared tumor tissue particles were placed in a tissue culture flask containing 10 mL of RPMI-1640 medium (unless otherwise stated, all reagents were purchased from Sigma, Taiwan) supplemented with 2 mg mL^−1^ collagenase-1. The tumor tissue particles were enzymatically digested at 37°C under shaking condition for approximately 20 hrs. After incubation, the digested suspension was filtered through a tea strainer to remove undigested tissue and subsequently through a 20 *μ*m pore sterile filter. The cancer cells in the filtrate were then washed three times with RPMI-1640 medium solution by repeated centrifugation in a centrifuge (1,800 rpm for 5 min) and resuspended in RPMI-1640 medium solution. The cell suspension thus obtained was assessed microscopically for cell number and cell viability using a fluorescent dye kit (LIVE/DEAD Viability/Cytotoxicity Kit L-3224, Molecular Probes) [9, 15]. After cell staining, briefly, the images of live (green) and dead (red) cells were captured using a confocal microscope (LSM 510 META, Zeiss, Germany). This was followed by an image analysis to evaluate cell viability [9]. Only cell preparations with cell viability >95% were then used. In addition, the purity of primary oral cavity cancer cells isolated was also evaluated microscopically using an EGFR fluorescent dye kit [26] and the subsequent image analysis.

### 2.3. The Metabolic Activity of Primary Oral Cavity Cancer Cells Cultured under Different Medium pH Conditions in the 2D, Spheroid, and 3D Cell Culture Models

Conventional cell-based chemosensitivity assays are normally performed using a static cell culture model which might not be able to provide a stable and thus well-defined culture condition for such assays. In order to investigate the extent to which the physiology (e.g., metabolic activity) of primary cancer cells was influenced by such an unstable culture condition (e.g., culture medium pH), we performed the following experiments. In this work, the freshly isolated oral cavity cancer cells with equal total cell number (5.7 × 10^3^) were cultured in 3 different culture models (i.e., 2D, 3D, and spheroid cell culture models) and under different medium pH (pH 6.6, 6.8, 7.0, 7.2, and 7.4) conditions using the perfusion microbioreactor system (Figure 1(a)). Briefly, 5.7 × 10^3^ primary oral cavity cancer cells were seeded on the bottom surface of the microbioreactor chamber for 2D culture (Figure 1). To achieve this, the bottom PDMS surface of chamber was treated with 0.01% fibronectin solution (1-hour immersion) to enhance cancer cell attachment. After loading cell suspension into the chamber, moreover, 4-hour time was given to allow cell sedimentation and attachment based on our preliminary test. In addition, the equal amount of cancer cells was encapsulated in collagen-alginate hydrogel (2.4% (w/v) Type I collagen and 0.2% (w/v) alginate) to form a 3D cell culture construct (*D*: 1.5 mm;* H*: 0.2 mm;* V*: 0.353 *μ*L), having a cell density of 1.62 × 10^7^ cells mL^−1^, for the perfusion 3D cell culture. For the spheroid cell culture, its preparation was based on the conventional spheroid formation method, namely, the hanging drop technique [27]. In this study, the volume of each hanging drop (cell density: 1.5 × 10^5^ cells mL^−1^) was 38 *μ*L.

After the 3 cancer cell culture models were prepared, perfusion cell culture was performed using the microbioreactor system (Figure 1(a)) for up to 2 days. In this work, the culture medium with different pH levels as aforementioned was continuously supplied to the microbioreactors at the flow rate of 15 *μ*L hr^−1^. During the culture period, the waste medium was collected for the measurement of lactate. In this study, the lactate produced and released into culture medium was measured using a Lactate Reagent Kit (Trinity Biotech Plc., Ireland) [6]. The assay was carried out as directed by manufacturer's instructions. A lactate solution at a concentration of 50~500 mg L^−1^ made from dissolving lactate sodium salt in deionized water (DI) water was used as standard. Moreover, the total number of cells after culturing was also explored by quantifying the DNA content of the cells [6].

### 2.4. Drug Chemosensitivity of Primary Oral Cavity Cancer Cells to Cisplatin under Perfusion 2D, Spheroid, 3D, and Tumor Tissue Culture Models

In this study, the anticancer drug chemosensitivity of the primary oral cavity cancer cells cultured in the 3 different models as aforementioned was evaluated. Briefly, the 3 cell culture models were prepared similarly to the descriptions in Section 2.3. In this work, the total cell numbers used in each cell culture models were 3 × 10^4^, in which the cell density of the 3D cell culture model was 7.6 × 10^6^ cells mL^−1^. For the 3D cell culture model, another case with higher cell density (8.5 × 10^7^ cells mL^−1^) used was also investigated. For comparison purpose, a tumor tissue culture model was also established, in which the prepared tumor tissue particles (Figure 2(b)) were directly cultured in the microbioreactor. In this work, the culture medium supplemented with the cisplatin at 3 different concentrations (0, 4, or 8 *μ*g mL^−1^) was supplied to the microbioreactors at the flow rate of 15 *μ*L hr^−1^. After 2-day culture, the cultured cells and tumors were assayed for cell viability using the Cell Counting Kit 8 (CCK-8) [28]. Apart from the quantitative evaluation, the viabilities of the oral cavity cancer cells cultured in the 3 different cell culture models and in the tumor culture model tested after treatment with different concentrations of cisplatin were observed microscopically using the fluorescent dye kit (LIVE/DEAD Viability/Cytotoxicity Kit L-3224, Molecular Probes) as aforementioned. For the 2D, 3D, and spheroid culture models, the samples were directly treated with fluorescent dye followed by fluorescence-based microscopic observation. For the tissue model, the cancer cells within the tissue sample cannot be easily treated with fluorescent dye and observed microscopically due to the dense tissue matrix. To tackle the issue, the drug treated tissue sample was then digested based on the method for primary cancer isolation (Section 2.2). The isolated cancer cells were then treated with fluorescent dye and observed microscopically. To avoid excessive cell aggregation during fluorescent dye treatment and microscopic observation, the obtained cell suspension was properly diluted.

### 2.5. Statistical Analysis

In this study, the data were presented as the mean ± the standard deviation from three separate experiments. For a given experiment, each condition was tested in triplicate. One-way ANOVA analysis with a statistical significance level of 0.05 was used to examine the effect of medium pH condition on the metabolic activities of primary cancer cells and the effect of cell culture models on the cell proliferation as well as the outcomes of chemosensitivity assays. The Tukey honestly significant difference (HSD)* post hoc* test was used to compare the differences between two conditions investigated when the null hypothesis of ANOVA analysis was rejected.

## 3. Results and Discussion

### 3.1. Perfusion Microbioreactor System for Cancer Cell-Based Chemosensitivity Assays

Animal cell cultures are widely used as* in vitro* cell-based models for various biological researches. However, the most commonly used cell culture model (e.g., static 2D monolayer cell culture) at present has several inherent limitations, mainly including the inability to precisely and faithfully probe real cellular responses to tested conditions. These are mainly because of its inability to create physiologically relevant culture environments and to precisely control and define extracellular conditions [6, 8]. For the latter, the manual culture medium replacement process in the conventional static cell culture practices normally leads to a fluctuating culture environment. Under this circumstance, for example, the nutrient, waste, tested drug, or pH level could vary with the periodic medium change process. Because the biological cells are fairly sensitive to extracellular environments [29], such unstable culture conditions might interfere the precise quantification of the cellular response to the specific culture condition (e.g., drug species or concentration) investigated.

**Figure 3:**
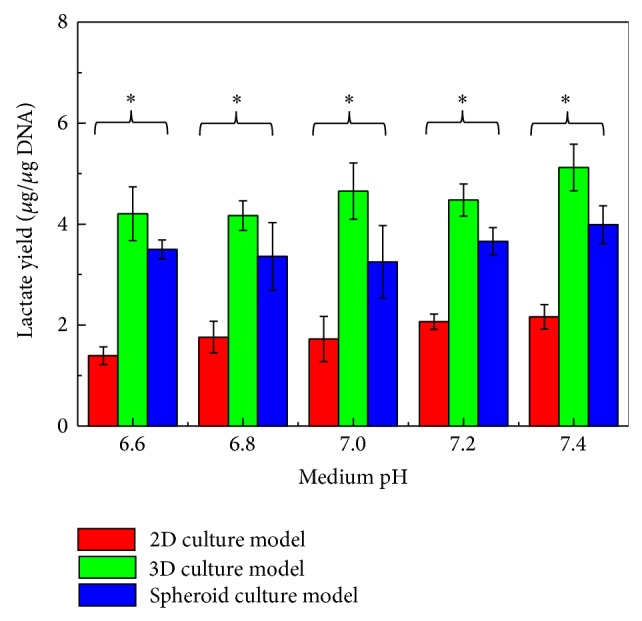
Evaluation of the metabolic activities of primary oral cavity cancer cells cultured in different medium pH conditions (pH 6.6, 6.8, 7.0, 7.2, and 7.4) and under 3 different cell culture models (2D, 3D, and spheroid culture models). The significant difference is expressed by a star (*P* < 0.05, ANOVA).

To explore the extent to which the physiology of primary cancer cells was influenced by such an unstable culture condition, experiments were carried out. In this work, the primary cancer cells were isolated from oral tumor tissues and were proved to have high cell viability (96 ± 2%) (Figure 2(c)) and purity (94 ± 4%) (Figure 2(d)). The effect of medium pH variations on the metabolic activity of primary oral cancer cells was used as a demonstration case. In this study, the variation range of medium pH (pH 6.6, 6.8, 7.0, 7.2, and 7.4) studied is within the medium fluctuation range normally occurring in a static cell culture practice. Moreover, it is a well-known fact that most cancer cells predominantly produce energy by a high rate of glycolysis followed by lactic acid fermentation in the cytosol [30, 31]. Therefore, the lactate produced by the cancer cells was used as an indication of metabolic activity in this study. Results (Figure 3) revealed that, under the same cell culture model studied, the metabolic activity of cells was significantly influenced by the medium pH variations (*P* < 0.05, ANOVA). This demonstration case might indicate that the fluctuating cell culture environments encountered in a normal static cell culture could affect the physiology of primary cancer cells. This could therefore hamper the precise quantification of the cellular responses to the specific drug condition tested. This technical problem can be solved out if a perfusion-based microbioreactor system is utilized, which has been previously proved to provide a stable culture condition due to the continuous nutrient supply and waste removal [6]. Because of microscale cell culture format, furthermore, the chemical gradient phenomenon existing in such a cell culture model is low so that the cell culture conditions within can be precisely defined and manipulated [8]. All these technical traits largely benefit a precise cell-based assay that could be currently impossible to achieve by using a conventional static cell culture model.

### 3.2. Effect of Cell Culture Models on the Metabolic Activities and Proliferation of Primary Oral Cavity Cancer Cells

2D, 3D, and spheroid cell culture models are widely used in various cancer cell-related studies [11–13, 18, 19, 23, 24]. In cell-based assays, however, one cannot merely extrapolate the data obtained from one cell culture model to another because the biochemical or biophysical microenvironments in the two situations could not be identical. Therefore, the data interpretation of cell-based assays would be challenging in terms of reconciling differences with data acquired through different cell culture models. Nevertheless, little is known about the effect of these cell culture models on the physiology of the cancer cells within the culture systems although some studies have tried to address the issue [9, 15]. To the best of our knowledge, however, most of these studies used cancer cell line as a research model for such investigations that could not be able to faithfully reflect the primary cancer cells' response to these cell culture formats. To more realistically address the biological issue, the influence of 2D, 3D, and spheroid cell culture models on the metabolic activity and cell proliferation of freshly isolated oral cavity cancer cells was explored in this study. Results (Figure 3) showed that the use of cell culture models had significant impact (*P* < 0.05, ANOVA) on the metabolic activity of primary oral cavity cancer cells under a given medium pH condition. The metabolic activities of cancer cells in the collagen-alginate-based 3D cell culture were statistically (*P* < 0.05) higher than those in the spheroid cell culture model, a cell aggregate-based 3D cell culture model. Within the experimental conditions investigated, moreover, the cancer cells in the conventional 2-D monolayer model had the lowest metabolic activity. Such metabolic activity is about 33–42%, and 39–56% of that in the 3-D, and spheroid cell culture models, respectively.

**Figure 4:**
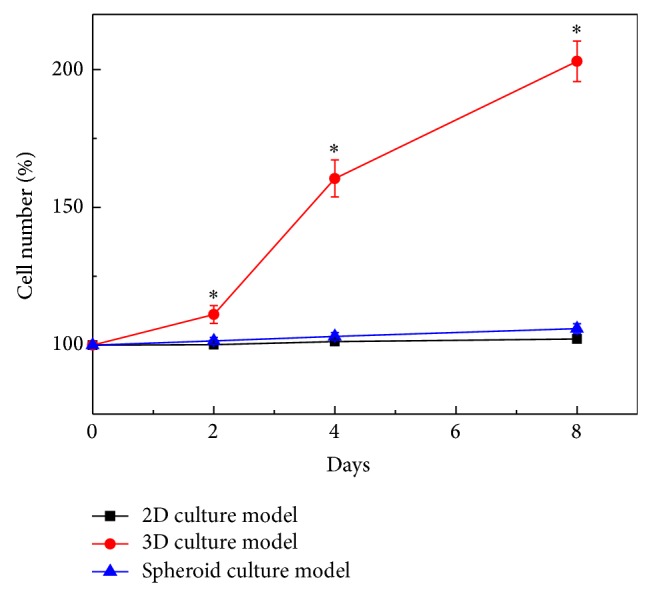
Proliferation curves of primary oral cavity cancer cells cultured in 3 different cell culture models (2D, 3D, and spheroid culture models). The significant difference is expressed by a star (*P* < 0.05).

**Figure 5:**
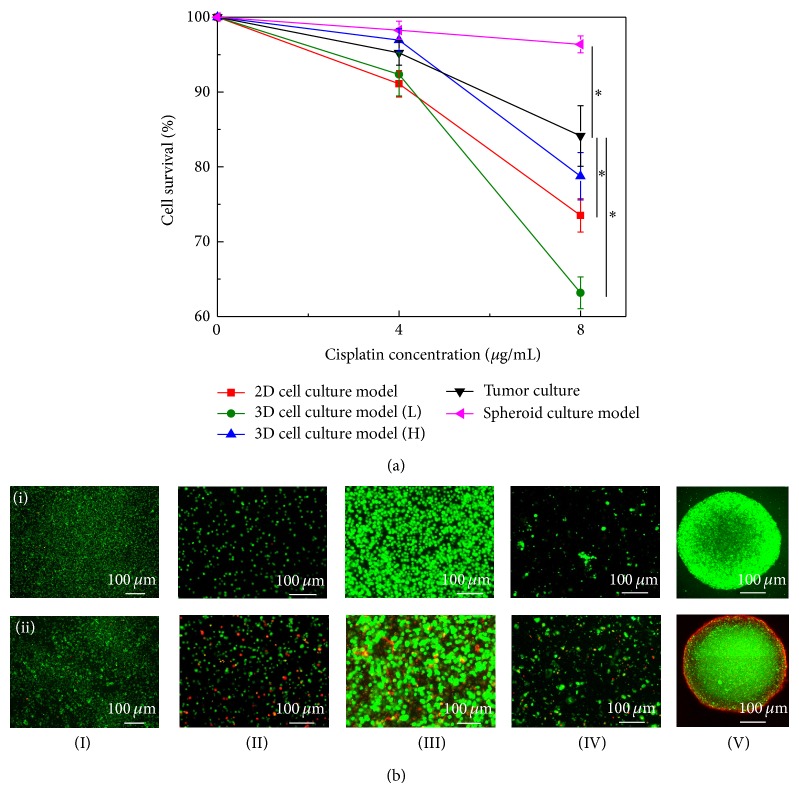
(a) Chemosensitivity evaluations of primary oral cavity cancer cells cultured in various cell culture models and treated with cisplatin at various concentrations; significant difference is expressed by a star (*P* < 0.05), (b) confocal microscopic observation of cell viability of cancer cells cultured in various cell culture models ((I): 2D culture model; (II): 3D culture model (low cell density); (III): 3D culture model (high cell density); (IV): tumor culture model; (V): spheroid culture model) and treated with cisplatin at the concentration of (i) 0 and (ii) 8 *μ*g/mL; green and red dots represent live and dead cells, respectively.

Regarding cell proliferation, results (Figure 4) revealed that the proliferation (%) of the primary cancer cells (the percentage of the total DNA content of cells at a particular time point over its initial DNA content) was significantly influenced by the choice of the cell culture model (*P* < 0.05, ANOVA). At days 2, 4, and 8 time points investigated, the proliferation of the primary cancer cells in the collagen-alginate-based 3D cell culture model was about 11–101% statistically higher than that in the 2D and spheroid-based cell cultures (*P* < 0.05). Within the experimental conditions explored, the proliferation of cancer cells in the 2D and spheroid culture models showed no statistical difference (*P* > 0.05). Overall, the above findings conflict with the previous cancer cell line-based study showing that the proliferation of cancer cells cultured in 2D monolayer format was significantly higher than that in a 3D culture model [15]. In this work, the low cell proliferation of the primary cancer cells cultured as 2D monolayer could be due to their inability to fully attach and spread on a 2D surface for cell proliferation within the time period explored. This phenomenon was also confirmed microscopically (Figure 5(b)-(I)). In this study, the PDMS surface was treated with 0.01% fibronectin to enhance cell attachment. This approach is the commonly used method to enhance cell attachment on a PDMS surface. Even prolonged time was given, and the cancer cells were observed just to adhere on the treated surface without full attachment. This phenomenon was also observed when the conventional multiwell microplates were used for such primary cancer cell culture (images not shown).

### 3.3. Effect of Cell Culture Models on the Chemosensitivity of Primary Oral Cavity Cancer Cells to Cisplatin

In this study, a two-day culture time was employed, based on the test time commonly used for 2D cell culture-based assays of the chemosensitivity of cancer cells toward anticancer drugs [32]. Moreover, it is a well-known fact that the results of tissue-level assays are much closer to the real outcomes than the cell-based assays. For comparison purpose, therefore, the results of the chemosensitivity assays based on the cell culture models tested were compared with that based on the tumor tissue-level chemosensitivity assays. For the 3D cell cultures studied, furthermore, 2 kinds of culture models with different cell density levels (7.6 × 10^6^ and 8.5 × 10^7^ cells mL^−1^, indicated as “*H*” and “*L,*” respectively, in Figure 5) were prepared for the chemosensitivity tests. For the higher cell density case, its cell density level (8.5 × 10^7^ cells mL^−1^) was same as that in the tumor tissue samples tested, based on our preliminary studies. It is not surprising that the results (Figure 5(a)) revealed that the cell survival percentage of primary cancer cells decreased upon increasing the dosage of cisplatin for each cell culture model tested. At the drug concentration of 8 *μ*g mL^−1^, the choice of the cell culture models had a significant impact on the chemosensitivity assay results (*P* < 0.05, ANOVA) (Figures 5(a) and 5(b)). For the cell culture-based assays explored, overall, the percentage of cell survival in the chemosensitivity assays based on the spheroid cell culture models was significantly higher than that in the high-cell density 3D and 2D cell culture models. In addition, the cell survivals in these 3 models were statistically higher than that based on the low-cell density 3D cell culture model. Cisplatin is particularly effective at killing cancer cells during their proliferation process [33]. The higher proliferation rates observed in the 3D cell culture model (Figure 4) are consistent with their accordingly lower cell survival (%) in the low-density 3D cell culture-based chemosensitivity assays (Figure 5(a)). The above relationship between cell proliferation and cell survival, however, could not explain the chemosensitivity difference between the assays based on the spheroid and 2D cell culture models because the cell proliferation in these 2 models revealed no significant difference (Figure 4). Compared with the lower cell survival percentage observed in the 2D culture-based chemosensitivity assays, the higher cell survival occurring in the spheroid culture-based chemosensitivity assays (Figure 5(a)) could be due to the mass transfer barrier existing in the compact cell aggregate system, by which the anticancer drug might not be able to effectively act on the cells within. This phenomenon was also observed microscopically (Figure 5(b)-(V)), in which the dead cancer cells (red dots) are mainly distributed around the surface of cell aggregate particle, whereas the cancer cells located inside still kept viable (green dots).

Regarding the comparison of the chemosensitivity outcomes based on the cell-based and tumor tissue-level assays, the following descriptions and discussions were based on the treatment of cisplatin at the concentration of 8 *μ*g mL^−1^ (Figures 5(a) and 5(b)). Results exhibited that the cell survival percentage in the spheroid culture-based assays was significantly higher than that in the tumor tissue-level tests (*P* < 0.05). This result could imply that the spheroid culture-based chemosensitivity assays might overestimate the resistance of primary cancer cells to the anticancer drug tested. This phenomenon, again, could be reasonably explained by the aforementioned mass transfer barrier effect because the cancer cells formed a cell aggregate particle, which was more compact than the native tumor tissue. In addition, the drug chemosensitivity of primary cancer cells in the tumor tissue-level and the high-cell density 3D culture models showed no statistical difference, indicating that the drug chemosensitivity of cancer cells in such 3D culture system was closer to that in the native tumor tissue. Moreover, the cell survivals in the 2D and low-cell density 3D culture-based assays were significantly lower (*P* < 0.05) than those in the tissue-level assays, which might imply that the use of these 2 cell culture models could overestimate the chemosensitivity of primary cancer cells to cisplatin. Unlike the 3D culture models with the same cell density (8.5 × 10^7^ cells mL^−1^) as the tumor tissue sample tested (namely, the high cell density 3D model), the high chemosensitivity of cancer cells to cisplatin occurred in the similar 3D culture model with lower cell density (7.6 × 10^6^ cells mL^−1^) could be due to the cell proliferation effect. Primary cancer cells in the low-cell density 3D environment could have more space to proliferate whereas their proliferation was inhibited under a high cell density condition. Therefore, the higher cell proliferation phenomenon occurring in the low-cell density 3D culture model could accordingly lead to a higher cytotoxic effect of cisplatin to the cancer cells. This speculation was also confirmed microscopically (Figures 5(b)-(II) and 5(b)-(III)). In order to find out the cellular response to the anticancer drug tested under different cell culture models, overall, the cells had to be inevitably arranged in different cell culture formats. In these situations, the influence of mass transport phenomenon cannot be perfectly ruled out. In this study, one of the technical advantages of using microscale cell culture model is its ability to minimize the chemical gradients existing in the cell culture system (e.g., cell culture construct). Further experiments (e.g., further miniaturized cell culture models using microfluidic technology) are required to possibly rule out the impact of mass transport phenomenon so as to more precisely investigate the real cellular response to the drug condition tested.

## 4. Conclusions

Cell cultures are widely used as* in vitro* cell-based models for various biological researches (e.g., drug chemosensitivity assays). The most commonly used cell culture models are static 2D monolayer cell culture models. However, the influence of fluctuating culture conditions occurring in such cell culture model on the physiology of the cultured cells is generally ignored. In this study, experiments showed that even minor environmental pH change could significantly affect the metabolic activity of cells, demonstrating the importance of a stable culture condition for such assays. To tackle this issue, a perfusion-based microscale cell culture model capable of providing a stable culture condition was adopted. In addition to the conventional 2D monolayer cell culture models, several new types of cell culture methods (e.g., spheroid or 3D culture models) were actively proposed. However, little is known about the impact of these cell culture models on the physiology and the drug chemosensitivity assay results of primary oral cavity cancer cells. To address the fundamental biological issues, experiments were carried out. Results revealed that the choice of cell culture formats (e.g., 2D, 3D, or spheroid culture models) might play an important role in the physiology (e.g., metabolic activity or cell proliferation) of the cultured cells. Also the use of different cell culture models could lead to different results of drug chemosensitivity assays. Compared with the similar test based on tumor tissue-level assays, the use of spheroid culture model could overestimate the drug resistance of cells to cisplatin, whereas the utilization of 2D and 3D culture model might overestimate the chemosensitivity of cells to such anticancer drug. In cell culture-based assays, thus, the extrapolation of experimental results from one cell culture format to another might lead to biases because the biochemical or biophysical microenvironments in the two situations could not be identical. Compared with the tumor tissue-level chemosensitivity assays, moreover, the 3D culture models with same cell density as that in tumor tissue samples showed comparable chemosensitivity assay results as the tumor tissue-level assays. As a whole, this study has provided some fundamental information regarding the impact of cell culture methods on the results of* in vitro* cell-based assays. All these pieces of information are of great importance for establishing a precise and faithful drug chemosensitivity assay.
